# A Case of Sublingual Ranula That Responded Successfully to Localized Injection Treatment with OK-432 after Healing from Drug Induced Hypersensitivity Syndrome

**DOI:** 10.1155/2016/6939568

**Published:** 2016-04-06

**Authors:** Kunio Yoshizawa, Akinori Moroi, Shuichi Kawashiri, Koichiro Ueki

**Affiliations:** ^1^Department of Oral and Maxillofacial Surgery, Division of Medicine, Interdisciplinary Graduate School of Medical and Engineering, University of Yamanashi, 1110 Shimokato, Chuo, Yamanashi 409-3898, Japan; ^2^Department of Oral and Maxillofacial Surgery, Kanazawa University Graduate School of Medical Science, 13-1 Takaramachi, Kanazawa, Ishikawa 920-8641, Japan

## Abstract

A ranula is a mucus retention cyst or pseudocyst caused by leakage of mucus from the sublingual gland and generally occurs in the oral floor. In addition, drug induced hypersensitivity syndrome (DIHS) is a rare but well-recognized serious adverse effect characterized by fever, skin rashes, generalized lymphadenopathy, hepatitis, and hepatosplenomegaly and oral stomatitis. This paper presents the first case of successfully treated sublingual ranula with localized injection of OK-432 after healing from drug induced hypersensitivity syndrome, which has previously been unreported in the literature. We present the case of a 38-year-old Japanese woman with sublingual ranula that responded successfully to localized injection treatment with OK-432 after healing from drug induced hypersensitivity syndrome. She was affected with cutaneous myositis and interstitial lung disease when she was 26 years old. At the age 34 years, she received additional oral treatment of diaminodiphenyl-sulfone due to deterioration of the cutaneous myositis, which resulted in drug induced hypersensitivity syndrome (DIHS) with severe oral stomatitis. Local injection of OK-432 to the ranula may be a very safe and useful treatment method even if the patient has a history of drug allergy and has connective tissue disease such as cutaneous myositis.

## 1. Introduction 

Drug induced hypersensitivity syndrome (DIHS) is a rare but well-recognized serious adverse effect characterized by fever, skin rashes, generalized lymphadenopathy, hepatitis, and hepatosplenomegaly and oral stomatitis [1, 2]. A ranula is a mucus retention cyst or pseudocyst caused by leakage of mucus from the sublingual gland and generally occurs in the oral floor. OK-432 (Picibanil®, Chugai Pharmaceutical Co., Tokyo, Japan), which is a lyophilized streptococcal preparation made from the low virulence Su strain of A-group* Streptococcus pyogenes* incubated with penicillin, is useful to treat ranula [3].

First, complete enucleation of the ranula by surgery is a challenge because its wall is so thin that it is liable to rupture during the procedure, rendering the surgery inadequate [4, 5]. Secondly, the risk of causing damage to the surrounding lingual nerve and Wharton's duct may increase because operative field is too narrow and adjoining to the tissue [6]. Considering the high rate of recurrence and potential complications of surgical management of a sublingual ranula, local injection of OK-432 is reasonable. Benefits of this therapy as compared with other procedures are summarized as follows. (1) With regard to cost performance, no hospitalization is required. (2) The treatment is painless and the time required for the procedure is brief, which means it can be well tolerated by children and nervous patients. (3) No local anesthesia is required during the procedure. (4) Nerve injury and cosmetic problems are avoided. (5) Secondary infection and hemorrhage are rare. (6) Recurrences are less frequent. (7) The response does not appear to extend beyond the cystic cavity, avoiding any difficulty or increased morbidity of subsequent surgeries [3, 7]. Many authors that have reported on the use of OK-432 injection to treat ranula have indicated it to be a very effective treatment for both intraoral and plunging ranulas. Fukase et al. [8] reported disappearance of the ranula after injection therapy in 97% of cases.

This paper presents the first case of successfully treated sublingual ranula with localized injection of OK-432 after healing from drug induced hypersensitivity syndrome, which has previously been unreported in the literature.

## 2. Case Report

**Figure 1:**
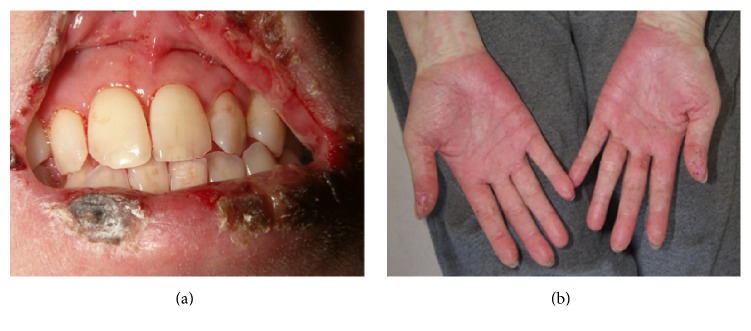
DIHS shows a systemic mucocutaneous disease. (a) Severe erosion with haphalgesia in the entire mouth. (b) Palmar lesion with reddening.

**Figure 2:**
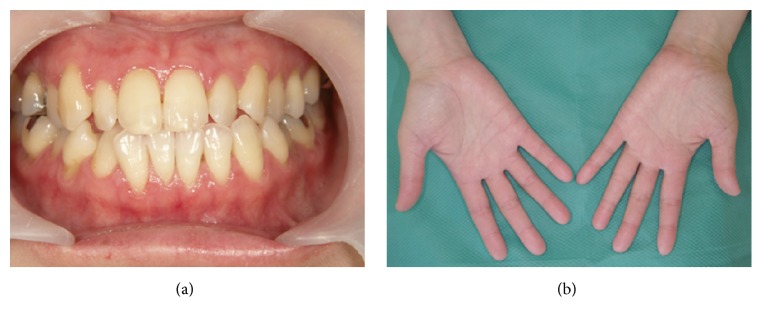
Healed state of the mouth and hand after treatment for DIHS.

We present the case of a 38-year-old Japanese woman with sublingual ranula that responded successfully to localized injection treatment with OK-432 (Picibanil, Chugai Pharmaceutical Co., Tokyo, Japan) after healing from drug induced hypersensitivity syndrome. She was affected with cutaneous myositis and interstitial lung disease when she was 26 years old. Therefore, she was treated with oral prednisolone 60 mg, and in 2008, at the age 34 years, she received additional oral treatment of diaminodiphenyl-sulfone (dapsone) (Lectisol®, Mitsubishi Tanabe Pharma Co., Osaka, Japan) due to deterioration of the cutaneous myositis. She received oral treatment of dapsone for a few days, which resulted in drug induced hypersensitivity syndrome (DIHS) with severe oral stomatitis. She presented with these symptoms on her first visit for oral and maxillofacial surgery (Figure 1). By replacing the offending drug with Lectisol and starting her on steroid pulse therapy, the DIHS and oral stomatitis were resolved (Figure 2).

**Figure 3:**
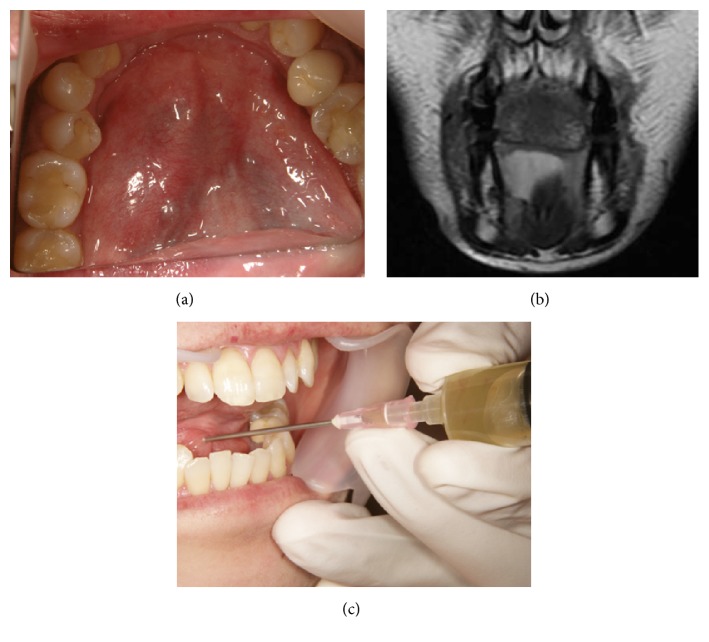
Appearance of the ranula at the first medical examination shows swelling of the anterior floor of the mouth (using mirror) (a). T2-weighted magnetic resonance images before treatment, showing the high-intensity areas in the right sublingual regions (b). The aspirated content from the cystic lesion was yellowish and mucinous (c).

**Figure 4:**
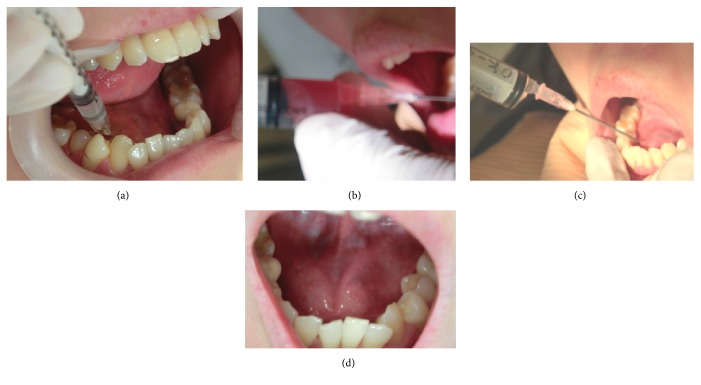
Procedure of first local injection of OK-432 solution (0.7 KE/0.3 mL) using a 27-G needle (a). The cyst showed no inflammation after the first procedure. Nine consecutive days later, we punctured the cyst using an 18 G needle, and the maximum possible amount of mucus (10 mL) was aspirated immediately (b). OK-432 solution (0.2 Klinische Einheit [KE] per millimeter; total 1 KE/5 mL) was then injected using the same needle (c). There was mild spontaneous pain and swelling with redness around the injected region over the center-line of the mouse at eight days after the second injection of OK432 (1 KE/5 mL) (d).

**Figure 5:**
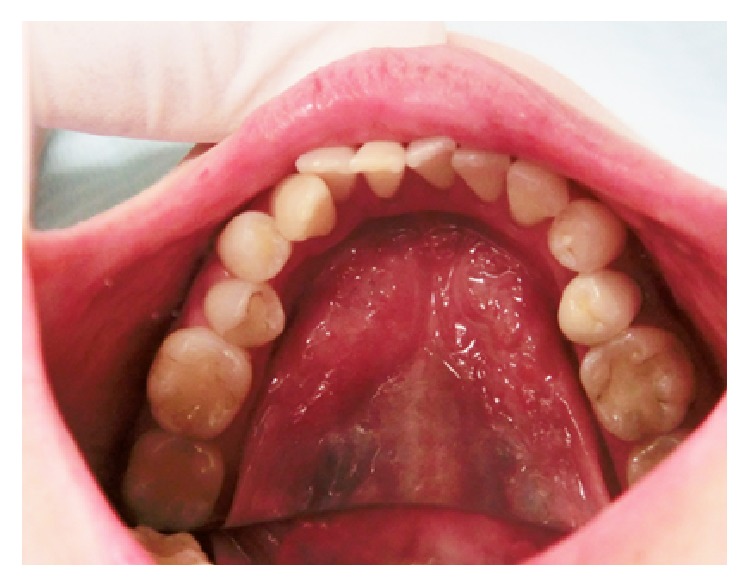
Ranula in the healed state following two treatments of local injection of OK-432.

However, in 2012 December at 38 years of age, she developed oral ranula and visited our department with sublingual swelling and pain as chief complaints. Swelling size was 50 m × 30 mm. T2 weighted magnetic resonance imaging (MRI) showed a high-intensity area in the right sublingual regions (Figure 3). The aspirated content from the cystic lesion was yellowish and mucinous. The findings confirmed the diagnosis of right sublingual ranula. The patient experienced reexpansion a few weeks after treatment by aspiration. After receiving a detailed explanation of available treatments including ranula excision, marsupialization, combined ranula and sublingual gland excision, and OK-432 intracystic injection, the patient selected the OK-432 treatment. We familiarized ourselves with the procedure for locally injecting OK-432 solution (0.7 KE/0.3 mL) using a 27-gauge (G) needle, which was inserted inside the cyst in February 2013, according to the method previously reported by Fukase et al. [9]. However, we could not confirm the effect of treatment in the absence of an inflammatory reaction for nine consecutive days. Thus, we additionally performed the following procedure which consisted of a series of punctures and injections. The cystic tumor was punctured using an 18 G needle, and the maximum possible amount of mucus (10 mL) was aspirated. OK-432 solution (0.2 Klinische Einheit [KE] per millimeter; 0.02 mg/mL, total 1 KE/5 mL) was then injected using the same needle, which was fixed inside the cyst, in order to avoid rapture or fluid spillage until the end of the procedure as reported previously by Ogita et al. [10] (Figure 4). On the day when the second injection was administered, the patient had mild inflammation and tenderness around the area of injection for 2 weeks and slight fever of 37.5–38°C for a few days. The ranula disappeared completely without severe complications such as interstitial lung disease and severe allergen reactions at 2 weeks after the second injection. No recurrence was observed during the 30-month postinjection period until presently (Figure 5).

## 3. Discussion

OK-432 exerts a sclerosing effect by immediately evoking inflammation into cystic space. When OK-432 is administered locally, inflammatory cells, such as neutrophils and monocytes, infiltrate the cyst and various cytokines, including IL-6, IL-8, IFN-*γ*, and TNF-*α*, are secreted [11]. Although fluid drainage and fibrotic adhesion induced by inflammation with OK-432 may be considered effective against abnormal exfiltration of saliva, the mechanism whereby OK-432 produces a better effect on ranula is not clearly understood.

In the present case, though we were concerned about the onset of severe complications due to her current and anamnestic history such as cutaneous myositis, interstitial lung disease, and DIHS, there was none. Dapsone is a drug of choice in the treatment of leprosy and other dermatoses including cutaneous myositis [1]. Dapsone hypersensitivity syndrome is a rare but well-recognized serious adverse effect characterized by fever, skin rashes, generalized lymphadenopathy, hepatitis, and hepatosplenomegaly and oral stomatitis [1, 12]. It may be caused by metabolites of dapsone forming haptens with the formation of anti-dapsone antibodies [13]. Thus, one may suggest that local injection of OK-432 was a secure and appropriate option as no adverse events such as drug allergy, which can be expected in cases of autoimmune skin disease and DIHS, occurred. Also, the findings in this case were similar to those of previous reports on OK-432 where serious adverse events and complications were avoided [8, 10, 14]. Moreover, we have reported this case report as the first case of successfully treated sublingual ranula with localized injection of OK-432 after healing from drug induced hypersensitivity syndrome. However, rigorous management after the injection procedure is important from the point view of Poldervaart et al. who reported that such treatment should always take place in treatment facilities specialized in the procedure for treating lymphatic malformations with OK-432 [14].

Leakage of the injected OK-432 could be a reason for the poor response to the first injection. Such leakage occurs due to increased swelling of the cyst as the inner pressure of the cyst may be too high. Another reason is that the amount of OK-432 (0.7 KE) was too low to evoke an adequate inflammatory reaction. Therefore, to avoid raising the internal pressure in the second treatment, the quantity of diluted OK-432 (5 mL) was adjusted to the half and allowed to be absorbed in the cystic cavity. This procedure was previously reported by Ohta et al. [11]. The intralesional fluid was highly viscous, so it was necessary to use a relative larger needle (18 G) to aspirate the fluid even though a smaller 27 G needle was preferred to avoid leakage of OK-432 from the cystic lesion. Our role is to seek a safe and effective method for administering OK-432 into an intraoral ranula.

## 4. Conclusions

We successfully treated sublingual ranula with localized injection of OK-432 after healing from drug induced hypersensitivity syndrome. Local injection of OK-432 to the ranula may be a very safe and useful treatment method without serious systemic adverse effect even if the patient has a history of drug allergy such as DIHS and has connective tissue disease such as cutaneous myositis.
